# Towards a generalized toxicity prediction model for oxide nanomaterials using integrated data from different sources

**DOI:** 10.1038/s41598-018-24483-z

**Published:** 2018-04-17

**Authors:** Jang-Sik Choi, My Kieu Ha, Tung Xuan Trinh, Tae Hyun Yoon, Hyung-Gi Byun

**Affiliations:** 1 Division of Electronics, Information and Communication Engineering, Kangwon National University (Samcheok), Kangwon-do, 25913 Republic of Korea; 2 Department of Chemistry, College of Natural Sciences, Hanyang University, Seoul, 04763 Republic of Korea

## Abstract

A generalized toxicity classification model for 7 different oxide nanomaterials is presented in this study. A data set extracted from multiple literature sources and screened by physicochemical property based quality scores were used for model development. Moreover, a few more preprocessing techniques, such as synthetic minority over-sampling technique, were applied to address the imbalanced class problem in the data set. Then, classification models using four different algorithms, such as generalized linear model, support vector machine, random forest, and neural network, were developed and their performances were compared to find the best performing preprocessing methods as well as algorithms. The neural network model built using the balanced data set was identified as the model with best predictive performance, while applicability domain was defined using k-nearest neighbours algorithm. The analysis of relative attribute importance for the built neural network model identified dose, formation enthalpy, exposure time, and hydrodynamic size as the four most important attributes. As the presented model can predict the toxicity of the nanomaterials in consideration of various experimental conditions, it has the advantage of having a broader and more general applicability domain than the existing quantitative structure-activity relationship model.

## Introduction

Quantitative structure-activity relationship (QSAR) model, which was developed by Corwin Hansch^1^, represents a technology aimed at providing estimates of many laboratory test results before the tests are conducted. The classic QSAR predicts biological activity related to various substances, based on a molecular structure which is represented as a vector of descriptors such as molecular graphs^2^, Simplified Molecular Input Line Entry Systems (SMILES)^3^, and International Chemical Identifiers (InChI)^4^.

During the last decade, various nanomaterials have been developed and extensively exploited in a variety of manufacturing processes for products and healthcare, including paints, filters, insulation, semiconductors, cosmetics, and biomedical devices^5^. In the case of nanomaterials, their physicochemical property, quantum-mechanical property, and different biological profile determine their interaction with living organisms^6–8^. It has been found that the inhalation, dermal contact and oral ingestion of nanomaterials could pose a risk to humans and environments^9,10^. Uptake of nanomaterials has been demonstrated to occur from epithelial and endothelial cells^11^. Therefore, the risk assessment for the nanomaterials has been considered as the important task in the nanotechnology field.

For nanomaterials, as most QSAR modelling activities are based on *in vivo* or *in vitro* data from particular experimental conditions (or obtained with different protocols)^12–17^, the applicability domain of the QSAR model is becoming narrower and more limited. Under this circumstance, the nanotechnologists have to always manually find specific QSAR models applicable to particular nanomaterials to screen their toxicity. Therefore, developing QSAR models having wider applicability domain is required so that the user such as nanotechnologists, nanomaterial manufacturers, and researcher easily use the models. In addition, the toxicity of nanomaterials varies according to the biological conditions such as assay method, cell type, cell line, cell origin, and cell species. This has driven our research toward developing a generalized toxicity prediction model.

Developing the generalized toxicity prediction model requires a standardized database containing comprehensive toxicity data of nanomaterials obtained using international protocols and good laboratory practice (GLP). In addtion, the quality and completeness of the toxicity data must be assessed and validated^18–21^.

Under such circumstances, Safe and Sustainable Nanotechnology (S2NANO), a research group in the Republic of Korea, built a S2NANO database (www.s2nano.org) including various experimental results related to nanomaterials obtained from different sources. The quality and completeness of collected data in the S2NANO database are assessed and validated using a physicochemical(PChem) score screening and nano-specific data gap filling method proposed by S2NANO^22^. The PChem score screening system evaluates the quality of physicochemical data while the nano-specific data gap filling method replaces missing values with manufacturer’s specifications and/or estimations.

For development of general models applicable to nanomaterial toxicity prediction, there has been an initial sign for developing QSAR models using quasi-SMILEs representing all conditions related to physicochemical properties and biological profiles such as toxic assay method, cell line, and so on^23–26^. The values of the physicochemical properties and biological profiles are coded to quasi-SMILES expressed in a simple syntactic sequence (i.e., character string). Quasi-QSAR model developed using the quasi-SMILES presented more generalized QSAR model than existing one. However, as the quasi-SMILES is made up using limited characters, it’s difficult efficiently to assign characters when the data contains many records and a wide range of data.

The solution is to use the appropriate pre-processing techniques to transform the comprehensive toxicity data into a numeric input metric suitable for generalized toxicity prediction models.

The object of this study is to develop a generalized toxicity prediction model for oxide nanomaterials using the S2NANO database, which consists of various experimental results for the nanomaterials. The model development methodology and results of the model development and validation were presented. In addition, effects of a few more preprocessing techniques were described in this paper.

## Materials and Methods

### Model development workflow

**Figure 1:**
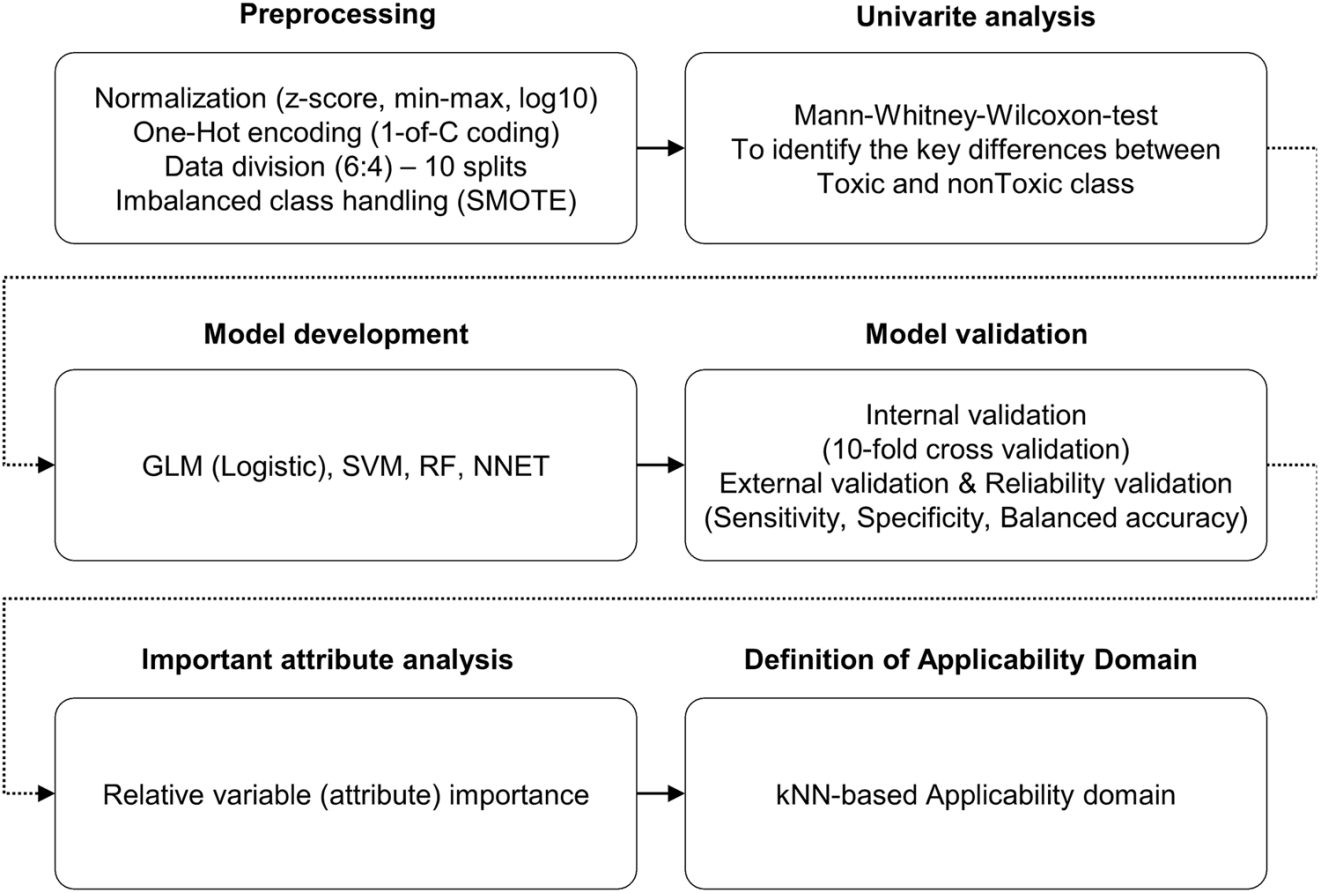
Model development workflow.

Figure 1 shows the workflow for the development of generalized QSAR models that are able to determine toxicity based on different biological conditions. A data set comprised of 574 observations was used in developing the models. Various methods were carried out in the preprocessing step and univariate analysis was conducted to identify the key differences between the toxic class and nonToxic class. After that, the data set was used for model development and validation. In addition, reliability of the developed model was validated using toxicity data obtained from the laboratory experiment conducted by S2NANO group. Four modelling algorithms, including the generalized linear model (GLM), support vector machine (SVM), random forest (RF), and neural network (NNET), were used for building the models, and their performance was evaluated via measures based on the confusion matrix. Analysis of relative optimal descriptors (attributes) was conducted. Finally, the k-nearest neighbors (kNN)-based applicability domain was defined in order to ensure the reliable prediction of the model which showed the best performance.

### Experimental Data

The data set with high PChem scores in the S2NANO database, which consists of various experimental results related to 7 oxide nanomaterials (ZnO, TiO_2_, SiO_2_, Fe_3_O_4_, Al_2_O_3_, CuO, and Fe_2_O_3_), was used for building a QSAR model (see S2NANO data for more information).

The PChem score representing data quality level is criteria which revised and expanded from existing evaluation criteria^18,19^ for assessing the quality of published experimental data on nanomaterials. The existing evaluation criteria include assessment as to whether or not toxicological data were obtained using international protocols (i.e., EU, EPA, FDA, OECD, etc.). In addition, the criteria evaluation method considers GLP. If data was generated in a laboratory that used GLP principles then, the quality of data should be better than that of data from a laboratory that was not working according to GLP principles. Using these reference criteria, the PChem scoring method evaluates the data quality taking into account the data source (experiment, manufacture, and article) and data method (the characterization methods for physicochemical properties: e.g., TEM, DLS, BET, etc.). The detailed criteria of PChem score are listed in Supplementary Table S1. High-PChem score means that the toxicity data was generated in a laboratory that used GLP principles and the physicochemical prosperities were characterized by widely recognized and acknowledged techniques (TEM, DLS, BET, etc. suggested by the OECD). As good quality input always result in the accurate prediction of properties, high quality data thoroughly evaluated by PChem scoring method was used in this paper.

15 attributes (physicochemical (PChem), quantum-mechanical (QM), and different biological profile (Tox)) listed in Table 1 were used as input descriptors.

**Table 1:** Attributes used in the model development.

PChem attributes		QM attributes	Tox attributes	
Core size (nm) 5.9–369	Surface charge (mV) −47.60–42.8	Formation enthalpy ΔHsf (eV) −17.35–−1.61	Assay method (AM) 8 types	Cell type (CT) (normal/cancer)
		Conduction band energy Ec (eV) −5.17–−1.51	Cell name (CN) 14 cells	Exposure time (hours) 3–72
Hydrodynamic size (nm) 74–1843	Specific surface area (m^2^/g) 7.0–576.23	Valence band energy Ev (eV) −11.12–−6.51	Cell species (CS) 3 species (Human, Hamster, Mouse)	Cell viability (%) (toxic or nontoxic) −3.87–151.11
		Electronegativity χMeO (eV) 5.67–6.19	Cell origin (CO) 8 types	Dose (mg/mL) 0–1440

Cell viability (%) was classified as either the toxic class or nonToxic class: if the cell viability was less than 50%, it was classified as the toxic class; otherwise, it was classified as the nonToxic class. The cell viability classified was used as an endpoint. As the data was collected from different sources, various assay methods, cell names, cell species, cell origins, and cell types were involved as nominal attributes in the data. The values of nominal attributes are listed in Supplementary Table S2.

## Results and Discussion

### Data preprocessing

Most QSAR models aim to predict endpoints related to nanomaterials under particular experimental conditions; however, the model which was developed in this paper, using the data extracted from S2NANO database, is aimed to predict endpoints for various nanomaterials under diverse experimental conditions. It is possible to predict endpoints under different experiment conditions if the value of each attribute is properly preprocessed according to their data characteristic. The used data consists of numeric attributes (PChem and QM) and nominal attributes (Tox); these attributes were normalized and encoded by taking their characteristic, data type (numeric or nominal), and model performance.

### Normalization for numeric attributes

As the measurement unit can affect the performance of the model, the data should be normalized or standardized^27,28^. Normalizing data is one step in addressing data that does not fit the model assumptions and is also used in coercing different variables to have similar distributions.

The numeric attributes in the used data were normalized via min-max, z-score, and log. The log transformation is often used for data which have positive skewness^29–31^. The normalization method, which is suitable for the data, was chosen by considering the distribution of each attribute. The skewness for each numeric attribute, a measure of symmetry in a distribution, is listed in Table 2. As attributes with the exception of hydrodynamic size, ΔHsf, and Ev have a right (positive) skewed distribution, those attributes were normalized using a log transformation.

**Table 2:** Skewness for each numeric attribute.

Attribute	Skewness (before)	Method	Skewness (after)
Core size	3.35	Log10	0.15
Surface charge	1.68	Log10	−0.04
Hydrodynamic size	0.46	Z-score	0.46
Surface area	3.24	Log10	0.34
ΔHsf	−0.35	Z-score	−0.35
Ec	1.75	Log10	0.09
Ev	−2.85	Min–max	−2.85
χMeO	2.28	Min–max	2.28
ET	1.39	Log10	−0.60
Dose	11.30	Log10	−0.14

The data of hydrodynamic size and ΔHsf were standardized via z-score because their skewed value is close to zero. As the skewness of Ev and χMeO was not improved after a log transformation, they were normalized by a min-max method. Most skewness for numeric attributes got closer to zero with the exception of Ev and χMeO after normalization. A zero value of skewness means that the tails on both sides of the mean balance out overall.

Additionally, the performance of the model according to different normalization methods including one considering the skewness was compared for each method in order to determine the optimal normalization method for each modelling algorithm. 10 data subsets divided randomly were normalized using min-max, z-score, log10, and combination (min-max, z-score, and log10) methods, and were used for building models. The models built were evaluated using 10-fold cross validation and a confusion matrix (the average value of balanced accuracy for 10 subsets was used). The results confirmed that log transformation is applicable to the GLM and the combination normalization method is suitable for the SVM and NNET algorithms as listed in Table 3. In particular, RF showed roughly similar performance regardless of the normalization method used.

**Table 3:** Model performance for each normalization method.

Model	Normalization method	True positive	False positive	False negative	True negative	Sensitivity	Specificity	Balanced accuracy
GLM	Min-Max	39	12	16	278	71%	96%	83%
	z-score	39	12	16	278	71%	96%	83%
	Log	46	11	9	279	84%	96%	90%
	combination	45	8	10	282	82%	97%	90%
SVM	Min-Max	28	6	27	284	51%	98%	74%
	z-score	29	7	26	283	53%	98%	75%
	Log	40	5	15	285	73%	98%	86%
	combination	41	5	14	285	75%	98%	86%
RF	Min-Max	45	5	10	285	82%	98%	90%
	z-score	44	5	11	285	80%	98%	89%
	Log	45	5	10	285	82%	98%	90%
	combination	45	5	10	285	82%	98%	90%
NNET	Min-Max	38	15	17	275	69%	95%	82%
	z-score	40	6	15	284	73%	98%	85%
	Log	43	8	12	282	78%	97%	88%
	combination	48	8	7	282	87%	97%	92%

### One-Hot encoding for nominal attributes

The categorical data must be converted to a numerical form because most prediction algorithms cannot operate on label data directly^32–37^; it may be converted using integer encoding and one-hot encoding. Integer encoding is used when the categorical attribute has a natural ordered relationship between each element, while one-hot encoding is used when the categorical attributes does not have an ordinal relationship. As the categorical attributes such as assay method, cell line, and so on do not have an ordinal relationship, they were encoded into dummy variables using the one-hot encoding. This encoding method allows the model to classify toxicity by considering various experimental conditions.

### Data division

A data set with a high PChem score was divided into 10 subsets randomly because the dangers of using the same data to both select and fit the model have been known for many years^38^. As more data subsets are used, the model performance converges in accordance with the law of large numbers^39^. The 10 subsets were divided into a training set and test set with a ratio of 60:40 for internal validation and external validation, respectively.

### Handling class imbalance problem

The class imbalance problem occurs when one of the classes has more samples than the other classes^40^. Most traditional classification algorithms can be limited in their performance on highly unbalanced data^41–45^. As the sample size of the two classes in the data used is highly imbalanced (toxic 16%, nonToxic 84%), SMOTE, which generates synthetic minority examples to over-sample the minority class^46^, was used to address the problem of class imbalance. The models for imbalanced data (ID) and balanced data (BD) were developed and compared in order to examine if the class balancing affects the predictive performance.

### Univariate analysis

Univariate analysis (Wilcoxon-Mann-Whitney-test (WMW)) was conducted to examine difference of the distribution between the toxic class and nonToxic class. As the data used are not normally distributed, a WMW test was carried out. WMW, which is used to assess not difference of means but the distribution of two independent groups, is the nonparametric alternative test to the independent sample t-test^47^. In this analysis, the high p-value indicates that there is a significant distribution difference between the two independent groups in a particular attribute. It means that the attribute with the high p-value in the WMW analysis could be considered as an important factor for the group discrimination.

The results of WMW are listed in Table 4. The attributes were sorted in descending order by p-value. ΔHsf has a significant mean difference between classes. In addition, it was confirmed that all attributes have a meaningful mean difference between classes (All p-values < 0.05). The important mean difference was identified in order of the QM attribute, Tox attribute, and PChem attribute.

**Table 4:** WMW analysis of attributes in data set.

Attribute	nonToxic		Toxic		z	p-value
	Mean rank	Sum of rank	Mean rank	Sum of rank		
ΔHsf	263.65	129188.50	426.63	35836.50	−8.93	4.28E-19
χMeO	306.09	149985.50	179.04	15039.50	7.03	2.07E-12
Dose	267.43	131041.50	404.57	33983.50	−7.02	2.23E-12
Surface area	307.31	150582.00	171.94	14443.00	6.92	4.37E-12
Ev	269.86	132233.50	390.38	32791.50	−6.60	4.04E-11
Exposure time	273.44	133986.50	369.51	31038.50	−5.23	1.70E-07
Ec	275.76	135124.50	355.96	29900.50	−4.39	1.11E-05
Core size	275.48	134987.00	357.60	30038.00	−4.21	2.58E-05
Surface charge	277.10	135778.00	348.18	29247.00	−3.64	2.70E-04
Hydrodynamic size	281.45	137910.50	322.79	27114.50	−2.11	3.45E-02

### Model development and validation

Four modelling algorithms, including GLM, SVM, RF, and NNET, were used for building the models, while the performance of models was evaluated by measures based on the confusion matrix.

GLM is extensions of traditional regression models that allow the mean to depend on the explanatory variables through a link function, and the response variable to be any member of a set of distributions called the exponential family^48^. GLM covers widely used statistical models, such as linear regression for normally distributed responses, logistic models for binary data, log-linear models for count data, and so on through its very general model formulation.

SVM is one of the most popular machine learning algorithms that can be employed for both classification and regression purposes^49^. In particular, SVM is more commonly used in classification problems. It performs classification by finding the hyperplane that maximizes the margin between the two classes. The vectors (cases) that define the hyperplane are the support vectors.

RF is an ensemble learning method, consists of an arbitrary number of simple trees, which are used to determine the final outcome^50^. For classification problems, the ensemble of simple trees votes for the most popular class.

NNET, usually called neural network, is a mathematical model and commonly used for classification in data science^51^. NNET is typically organized in layers such as input layer, hidden layer, and an output layer. An input pattern is applied to the input layer and its effect propagates, layer by layer, through the network until an output is produced. NNET is trained using optimization techniques like gradient descent with consideration for error between target and the output.

Confusion matrix, also known as an error matrix, is a specific table layout that allows visualization of the performance of an algorithm, typically a supervised learning one^52^. Each row of the matrix represents the instances in a predicted class while each column represents the instances in an actual class (or vice versa). Various measures, such as accuracy, sensitivity, specificity, and precision, are derived from the confusion matrix. This matrix is often used to describe the performance of a classification model.

The models were developed using R caret packages^53^. Basic measures such as balanced accuracy, sensitivity, and specificity from the confusion matrix, where toxic class is a positive instance and nontoxic class is a negative instance, were used. Accuracy computed from a confusion matrix is not a reliable measure for the real performance of a classifier, because it will yield misleading results if the data set is unbalanced. Therefore, the balanced accuracy considering both sensitivity and specificity were used.

The training set and test set were used for the model development, internal validation, and external validation. The validation results for 10 splits were averaged. In addition, toxicity data generated from an experiment conducted by S2NANO research group was used for the reliability validation of a developed model which showed the best performance.

### Internal validation

The commonly used k-fold cross validation technique is used for evaluating predictive models^54,55^. 10-fold cross validation is commonly used^56^. In particular, this validation method is used to avoid the overfitting problem and to estimate the general performance of model^57,58^. 10-fold cross validation was used for internal validation of models built using imbalanced and balanced data from the training data (60% of total data). The models built using the balanced training data showed better accuracy than the models built using the imbalanced training data. The basic measures of the confusion matrix for internal validation are listed in Table 5. The best results in terms of the basic measures were obtained from the NNET model built using balanced training data. Accuracy was improved on all models developed using the balanced training data.

**Table 5:** Internal validation result.

Model	Training data	True positive	False positive	False negative	True negative	Sensitivity	Specificity	Balanced accuracy
GLM	ID	46	11	9	279	84%	96%	90%
	BD	255	30	20	245	93%	89%	91%
SVM	ID	41	5	14	285	75%	98%	86%
	BD	269	5	6	270	98%	98%	98%
RF	ID	45	5	10	285	82%	98%	90%
	BD	268	2	7	273	97%	99%	98%
NNET	ID	48	8	7	282	87%	97%	92%
	BD	272	6	3	269	99%	98%	98%

### External validation

Table 6 lists the results of external validation for the models built using the imbalanced and balanced data from the test data (40% of total data). Most models built using the balanced data showed better balanced accuracy, with the exception of the GLM model. The best results in terms of the basic measures were obtained from the NNET model built using balanced training data, as was the case for internal validation.

**Table 6:** External validation result.

Model	Training data	True positive	False positive	False negative	True negative	Sensitivity	Specificity	Balanced accuracy
GLM	ID	25	6	4	194	86%	97%	92%
	BD	26	21	3	179	90%	90%	90%
SVM	ID	22	5	7	195	76%	98%	87%
	BD	25	10	4	190	86%	95%	91%
RF	ID	24	3	5	197	83%	99%	91%
	BD	25	9	4	191	86%	96%	91%
NNET	ID	23	4	6	196	79%	98%	89%
	BD	27	13	2	187	93%	94%	93%

There was a trade-off between sensitivity and specificity. In general, as the minority samples (toxic class) rarely occur but very important, the classification model should be sensitive to the minority samples than majority them.

### Reliability validation

For reliability validation of the developed model, the toxicity data including 144 rows generated from an experiment conducted by S2NANO research group was used as the validation set. A549 and BEAS-2B cell lines were exposed to four oxide nanoparticles (SiO_2_, ZnO, TiO_2_, and Fe_3_O_4_) with various physicochemical properties with respect to the core size, hydrodynamic size, and surface charge. The exposure time was 24 hours. The concentration of nanomaterials ranged from 0 to 100 ppm. MTS and CCK-8 assays were used to measure the cell viability of the A549 and BEAS-2B cell lines, with the results expressed as a percentage compared with control samples. A data row was labeled “toxic” if the viability percent was less than 50%; otherwise, it was considered “nonToxic”. After labeling, it was confirmed that the number of the toxic and nontoxic class was 14 and 130, respectively.

After the preprocessing step for the toxicity data, the data was used for the reliability validation of the developed NNET model. The validation results are listed in Table 7 (see S2NANO reliability validation data for more information).

**Table 7:** Reliability validation result.

True positive	False positive	False negative	True negative	Sensitivity	Specificity	Balanced accuracy
9	8	5	122	64%	93%	79%

The model showed good prediction result. It implies that the developed model could be considered as a more generalized predictive model, which is capable of predicting the toxicity label for the nanomaterials with consideration for the diverse experimental conditions.

In the developed model, the ZnO nanomaterials with low ΔHsf value were classified to the toxic class as its dose was increased. The model was more sensitive to the dose change of ZnO in the A549 cell line than BEAS-2B cell line. In contrast, the other oxide nanomaterials such as SiO_2_, TiO_2_, and Fe_3_O_4_ with relatively high ΔHsf value were classified to nontoxic class.

The range of attributes changes according to PChem score. If data set with the high PChem score is used, the range of attributes is reduced. In contrast, the range is extended when dataset with the over medium PChem score is used. Because of this characteristic, lesser/bigger range of attributes was not considered in the models.

It may be possible to estimate the toxicity of the lesser size using extrapolation, the process of estimating, beyond the original observation range. If the discrimination threshold of the developed QNTR models was well adjusted, the extrapolation of the lesser or greater size may be valid.

The toxicity data for the reliability validation of the developed model includes the toxicity results of the lesser hydrodynamic size (69.1 nm and 45.6 nm) of Fe_3_O_4_ nanomaterials. After the reliability validation with the toxicity data, it confirmed that the developed model correctly classified a toxic class of the toxicity data. However, the extrapolation is subject to greater uncertainty and a higher risk of producing meaningless results. Therefore, the applicability domain of the model was defined to avoid such risks.

### Important attribute analysis

Variable (attribute) importance can be relatively measured and quantified based on information obtained from the models. The advantage of measuring the importance based on built model information is that it is more closely tied to the model performance and it may be able to incorporate the correlation structure between the predictors into the importance calculation^59^. The analysis of relative attribute importance for the built NNET model was carried out using a varimp function of caret package supported in R software^60^. The importance is measured based on weights between layers in the NNET model.

The results of important attribute analysis are presented in Table 8. Dose, ΔHsf, Exposure time, and Hydrodynamic size were relatively identified as important attributes when compared to the other attributes; this means that they acted as important attributes in classifying materials as either toxic or nonToxic. This result is similar to the previous result of MWM analysis.

**Table 8:** Relative importance.

Attribute	Relative importance
Dose	11.10
ΔHsf	7.34
Exposure time	5.33
Hydrodynamic size	4.43
Ec	3.66
Surface area	3.58
Core size	3.57
Cell species	2.88
xMeO	2.44
Cell type	2.44
Surface charge	1.90
Assay method	1.82
Ev	1.45
Cell name	1.33
Cell origin	1.06

Dose and ΔHsf were identified as important attributes in both analyses. That is, they play an important role in determining toxic label. In contrast, the exposure time and hydrodynamic size, that their importance was lower in the Univariate analysis, were considered as an important factor in the important attribute analysis based on information of the developed model. The toxicity data set for various nanomaterials under diverse experimental conditions were used in the model development. The effects and heterogeneities associated with the diverse experimental conditions were not considered in the Univariate analysis. On the other hand, the weights to be used for computing the relative importance for each attribute are adjusted with consideration for the diverse experimental conditions during the model training process. It indicates that the exposure time and hydrodynamic size are considered as an important attribute in the real situation. This result implies that the important attributes analysis provides more reliable results than the Univariate analysis in case that dataset including diverse environment conditions (e.g., cell line, assay method, etc.).

The importance of attributes were relatively measured based on built model information such as weight adjusted during model training. In the real scenario, the cell origin is an important attribute contributing to the change of the toxicity. However, the relative importance of the cell origin was measured low in the analysis. The result does not mean that the cell origin does not affect the toxicity change. The model cannot precisely predict the toxicity without the descriptive attributes such as cell origin, cell species, cell type, cell name, and assay method. The model predicts the toxicity of nanomaterial using all available information from the whole attributes. The descriptive attributes (nominal attributes) were coded to dummy variables. The dummy variables act like ‘switches’ that turn various parameter on and off in the developed model. The dummy variable which for some observation has a value of 0 will cause that variable’s coefficient to have no role in influencing the dependent variable, while when the dummy takes on a value 1 its coefficient acts to alter the intercept. Although the relative importance of the descriptive attributes was measured low, the descriptive attributes actually play an important role in the toxicity classification of the model.

### Definition of the applicability domain

The Organization for Economic Co-operation and Development (OECD) has recommended that for the application of validated QSAR models to the prediction of new data points, there is a strict requirement of defining the applicability domain (AD) according to Principle 3^61,62^. The AD is widely understood to express the scope and limitations of a model, i.e. the range of chemical structures for which the model is considered to be applicable^63^. Generally, the training set is used to define the AD with a range-based method, geometric methods, distance-based methods and probability density distribution-based method^64^. In the case of ANN-based classification models, the AD can be defined based on Euclidean distance (ED) metrics^65^.

As the best predictive performance was identified in the NNET model built using the balanced data set, kNN-based AD using ED metrics was defined. A new compound will be predicted by the model^66–68^ if and only if:

$${D}_{i}\le \, < {D}_{k}\,\, > +{\rm{Z}}\times {s}_{k};$$

where <*D*_*k*_> is the average Euclidian distance between each compound of the training set and its k nearest neighbors in the descriptors space, *s*_*k*_ is the standard deviation of the distances between each compound of the training set and its k nearest neighbors in the descriptors space, and Z is an empirical parameter (0.5 by default). For each test compound i, the distance *D*_*i*_ is calculated as the average of the distances between i and its k nearest neighbors in the training set.

The value of k was chosen as the square-root of the number of training patterns. As 10 NNET models for 10 subsets were built, the AD for each NNET model was defined and the average values for <*D*_*k*_>, *s*_*k*_, and Z were calculated in turn; in particular, the preferable Z value was selected by increasing the Z value from 0.1 to 2.4 in increments of 0.1 and identifying the value with the highest accuracy for the test set. The results are listed in Table 9. The average preferable Z value was identified as 0.77; this means that *D*_*i*_ for the new compound should be less than a cutoff value, (<*D*_*k*_> + 0.77 × *s*_*k*_), so that the new compound will be reliably predicted by the model.

**Table 9:** Applicability domain of the best model.

split	sensitivity	specificity	Balanced Accuracy	***k***	***<D***_***k***_>	***s***_***k***_	**Z**
1	91%	94%	93%	22	1.39	0.79	2.10
2	88%	92%	90%	22	1.40	0.82	0.80
3	95%	98%	96%	23	1.40	0.80	0.40
4	94%	95%	95%	24	1.42	0.79	0.50
5	82%	97%	89%	22	1.40	0.81	0.30
6	96%	97%	97%	21	1.35	0.79	0.40
7	100%	94%	97%	22	1.40	0.79	0.50
8	86%	95%	90%	22	1.39	0.78	1.60
9	95%	97%	96%	25	1.47	0.80	0.60
10	100%	96%	98%	25	1.49	0.80	0.50
avg.	93%	96%	94%	22.8	1.41	0.80	0.77

## Conclusions

We developed a generalized QSAR model using a dataset with a high PChem score in the S2NANO database, which includes various experimental results. Various preprocessing techniques and modelling algorithms were used for model development. In addition, the analysis of relatively important attributes based on model information was performed. Finally, the kNN-based AD region was set up. As the proposed model can predict the toxicity of the nanomaterials in consideration of various experimental conditions, it has the advantage of having a broader and more general AD than the existing QSAR model. The results of this paper also indicate that preprocessing techniques appropriate to the characteristic of data should be applied for the generalized QSAR model.

The existing QSAR models can only predict the toxicity endpoint of nanomaterials under specific experiment condition. In contrast, the developed model can predict the toxicity endpoint (toxicity class) under the various experimental conditions conducted according to the international protocol and GLP. It allows the nanotechnologists as well as nanomaterial manufacturers, and researcher to obtain various toxicity results through one prediction model. Therefore, it enables the efficient utilization of the developed model.

The model development workflow presented in this paper can be considered a new methodology to develop a generalized QSAR model using a database containing various toxicity experimental results. Several databases exist that are relevant for engineered nanomaterials (ENM) toxicity assessment such as eNanoMapper, NanoMaterialRegistry, and Nanoparticle Information Library. The development of a generalized model for these databases using this methodology is expected to contribute to the application and utilization of QSAR.

## Electronic supplementary material

Supplementary infomation

S2NANO data

S2NANO reliability validation data

## References

[1] Randić M (1991). Novel graph theoretical approach to heteroatoms in quantitative structure—activity relationships. Chemom. Intell. Lab. Syst..

[2] McNaught, A. D. & Wilkinson A. *Compendium of Chemical Terminologyb* (ed. McNaught, A. D. & Wilkinson A.) 951 (Blackwell Science, 1997).

[3] Weininger D (1988). SMILES, a chemical language and information system. 1. Introduction to methodology and encoding rules. J. Chem. Inf. Model..

[4] Heller S, McNaught A, Stein S, Tchekhovskoi D, Pletnev I (2013). In ChI-the worldwide chemical structure identifier standard. J. Cheminform..

[5] Buzea C, Pacheco II, Robbie K (2007). Nanomaterials and nanoparticles: sources and toxicity. Biointerphases.

[6] Fadeel, B., *Handbook of safety assessment of nanomaterials: from toxicological testing to personalized medicine* (ed. Fadeel, B.) 222 (CRC Press, 2014).

[7] Aillon KL, Xie Y, El-Gendy N, Berkland CJ, Forrest ML (2009). Effects of nanomaterial physicochemical properties on *in vivo* toxicity. Adv. Drug Deliv. Rev..

[8] Gajewicz A, Puzyn T, Rasulev B, Leszczynska D, Leszczynski J (2011). Metal oxide nanoparticles: size-dependence of quantum-mechanical properties. Nanosci. Nanotechnol. Asia..

[9] Ray PC, Yu H, Fu PP (2009). Toxicity and environmental risks of nanomaterials: challenges and future needs. Journal of Environmental Science and Health Part C..

[10] Shakeel M (2016). Toxicity of nano-titanium dioxide (TiO 2-NP) through various routes of exposure: a review. Biological trace element research..

[11] Claudio R., Rinaldo B., & John A. K. Critical care nephrology (ed. Claudio R., Rinaldo B., & John A. K.) 1110–1116 (*Elsevier Health Sciences*, 2009).

[12] Puzyn T (2011). Using nano-QSAR to predict the cytotoxicity of metal oxide nanoparticles. Nat. Nanotechnol..

[13] Liu R (2011). Classification NanoSAR development for cytotoxicity of metal oxide nanoparticles. Small..

[14] Liu R (2013). Development of structure–activity relationship for metal oxide nanoparticles. Nanoscale..

[15] Liu R (2013). Nano-SAR Development for Bioactivity of Nanoparticles with Considerations of Decision Boundaries. Small..

[16] Singh KP, Gupta S (2014). Nano-QSAR modeling for predicting biological activity of diverse nanomaterials. RSC Adv..

[17] Pan Y (2016). Nano-QSAR modeling for predicting the cytotoxicity of metal oxide nanoparticles using novel descriptors. RSC Adv..

[18] Lubinski L (2013). Evaluation criteria for the quality of published experimental data on nanomaterials and their usefulness for QSAR modelling. SAR. QSAR. Environ. Res..

[19] Klimisch HJ (1997). A systematic approach for evaluating the quality of experimental toxicological and ecotoxicological data. Regulatory toxicology and pharmacology..

[20] Panneerselvam S, Choi S (2014). Nanoinformatics: emerging databases and available tools. Int. J. Mol. Sci..

[21] Marchese Robinson RL (2016). How should the completeness and quality of curated nanomaterial data be evaluated?. Nanoscale..

[22] My, K. H., *et al*. Toxicity Classification of Oxide Nanomaterials: Effects of Data Gap Filling and PChem Score-based Screening Approaches. *Scientific Reports.* **8**, 3141 (2018). 10.1038/s41598-018-21431-9 PMC5816655 29453389

[23] Manganelli S (2016). QSAR model for predicting cell viability of human embryonic kidney cells exposed to SiO2 nanoparticles. Chemosphere.

[24] Manganelli S (2016). QSAR Model for Cytotoxicity of Silica Nanoparticles on Human Embryonic Kidney Cells1. Materals Today: Proceedings..

[25] Toropova A (2015). A quasi-QSPR modelling for the photocatalytic decolourization rate constants and cellular viability (CV%) of nanoparticles by CORAL. SAR QSAR Environ. Res..

[26] Toropova A, Toropov A (2017). Nano-QSAR in cell biology: Model of cell viability as a mathematical function of available eclectic data. J. Theor. Biol..

[27] Han, J., Kamber, M. & Pei, J. *Data mining: concepts and techniques* 113–115 (Elsevier, 2011).

[28] Mitsa, T. *Temporal data mining* 25–26 (CRC Press, 2010).

[29] Bland JM, Altman DG (1996). Transformations, means, and confidence intervals. BMJ..

[30] Osborne JW (2010). Improving your data transformations: applying the box-cox transformation. Pract. Assess. Res. Eval..

[31] Buzsáki G, Kenji M (2014). The log-dynamic brain: how skewed distributions affect network operations. Nat. Rev. Neurosci..

[32] Cortez, P. & Morais, A. A data mining approach to predict forest fires using meteorological data, http://www.dsi.uminho.pt/~pcortez/fires.pdf (2007).

[33] Cortez, P. & Silva, A. Using data mining to predict secondary school student performance in *Proceedings of 5th Future Business Technology Conference (FUBUTEC 2008)* (ed. Brito, A. & Teixeira, J.) 5–12 (EUROSIS, 2008).

[34] Bengio, Y. & Bengio, S. Modeling high-dimensional discrete data with multi-layer neural networks. Advances in Neural Information Processing Systems, 400–406 (2000).

[35] Garson, G. D. *Neural networks: an introductory guide for social scientists* (Sage, 1998).

[36] Moeyersoms, J. & Martens, D. Data mining tip: how to use high-cardinality attributes in a predictive model https://www.kdnuggets.com/2016/08/include-high-cardinality-attributes-predictive-model.html (2016)

[37] Moeyersoms J, Martens D (2015). Including high-cardinality attributes in predictive models: a case study in churn prediction in the energy sector. Decis. Support. Syst..

[38] Faraway JJ (1998). Data splitting strategies for reducing the effect of model selection on inference. Comput. Sci. Stat..

[39] Diez, D. M., Barr, C. D. & Cetinkaya-Rundel, M. *OpenIntro statistics* 77–78 (CreateSpace, 2012).

[40] Longadge R, Dongre S, Malik L (2013). Class imbalance problem in data mining review. Int. J. Comput. Sci. Netw..

[41] Tang Y, Zhang Y-Q, Xhawla NV, Krasser S (2009). SVMs modeling for highly imbalanced classification. IEEE. Trans. Syst. Man. Cybern. B. Cybern..

[42] Chen JJ, Tsai CA, Young JF, Kodell RL (2005). Classification ensembles for unbalanced class sizes in predictive toxicology. SAR. QSAR. Environ. Res..

[43] Newby D, Freitas AA, Ghafourian T (2013). Coping with unbalanced class data sets in oral absorption models. J. Chem. Inf. Model..

[44] Capuzzi, S. J., *et al*. QSAR Modeling of Tox21 Challenge Stress Response and Nuclear Receptor Signaling Toxicity Assays. *figshare*, 10.3389/fenvs.2016.00003 (2016).

[45] Zakharov AV, Peach ML, Sitzmann M, Nicklaus MC (2014). QSAR modeling of imbalanced high-throughput screening data in PubChem. J. Chem. Inf. Model..

[46] Chawla NV, Bowyer KW, hall LO, Kegelmeyer WP (2002). SMOTE: synthetic minority over-sampling technique. J. Artif. Intell. Res..

[47] Mann HB, Whitney DR (1947). On a test of whether one of two random variables is stochastically larger than the other. Ann. Math. Stat..

[48] Olsson, U. Generalized linear models. *An applied approach. Studentlitteratur*. Lund (2002).

[49] Meyer D, Technikum Wien FH (2001). Support vector machines. R News..

[50] Liaw A, Wiener M (2002). Classification and regression by randomForest. R News..

[51] Haykin S, Network N (2004). A comprehensive foundation. Neural networks..

[52] Stehman SV (1997). Selecting and interpreting measures of thematic classification accuracy. Remote sensing of Environment..

[53] Kuhn M (2008). Caret package. J. Stat. Softw..

[54] Kohavi R (1995). A study of cross-validation and bootstrap for accuracy estimation and model selection. IJCAI..

[55] Refaeilzadeh, P., Lei, T. & Liu, H. Cross-validation in *Encyclopedia of database systems* (ed. Iu, L. & Özsu, M. T.) 532–538 (Springer US, 2009).

[56] McLachlan, G., Do, K. -A. & Ambroise, C. *Analyzing microarray gene expression data* Vol. 422 (John Wiley & Sons, 2005).

[57] Bengio Y, Grandvalet Y (2004). No unbiased estimator of the variance of k-fold cross-validation. J. Mach. Learn. Res..

[58] Arlot S, Celisse A (2010). A survey of cross-validation procedures for model selection. Stat. Surv..

[59] Ibrahim OM (2013). A comparison of methods for assessing the relative importance of input variables in artificial neural networks. J. Appl. Sci. Res..

[60] Kuhn, M. Variable importance using the caret package http://www.icesi.edu.co/CRAN/web/packages/caret/vignettes/caretVarImp.pdf (2012).

[61] Organisation for Economic Co-operation and Development. Guidance Document on the Validation of (Quantitative) Structure-Activity Relationship [(Q) SAR] Models. 32–40 (OECD Publishing, 2014).

[62] Roy K, Kar S, Ambure P (2015). On a simple approach for determining applicability domain of QSAR models. Chemom. Intell. Lab. Syst..

[63] Netzeva TI (2005). Current status of methods for defining the applicability domain of (quantitative) structure-activity relationships. The report and recommendations of ECVAM Workshop 52. Altern. Lab. Anim..

[64] Sahigara F (2012). Comparison of different approaches to define the applicability domain of QSAR models. Molecules..

[65] Fjodorova N, Novič M, Roncaglioni A, Benfenati E (2011). Evaluating the applicability domain in the case of classification predictive models for carcinogenicity based on the counter propagation artificial neural network. J. Comput. Aided. Mol. Des..

[66] Tropsha A (2010). Best practices for QSAR model development, validation, and exploitation. Mol. Inform..

[67] Zheng W, Tropsha A (2000). Novel variable selection quantitative structure−property relationship approach based on the k-nearest-neighbor principle. J. Chem. Inf. Comput. Sci..

[68] Tropsha A, Gramatica P, Gombar VK (2003). The importance of being earnest: validation is the absolute essential for successful application and interpretation of QSPR models. Mol. Inform..

