# Supplementary material for: Towards a generalized toxicity prediction model for oxide nanomaterials using integrated data from different sources
Source: Sci Rep. 2018 Apr 17;8:6110. doi: 10.1038/s41598-018-24483-z (PMC5904177; doi:10.1038/s41598-018-24483-z)
Supplement: Supplementary file 1 — Supplementary infomation [file 41598_2018_24483_MOESM1_ESM.docx]

Towards a generalized toxicity prediction model for oxide nanomaterials using integrated data from different sources

Jang-Sik Choi^1^, My Kieu Ha^2^, Tung Xuan Trinh^2^, Tae-Hyun Yoon^2^ and Hyung-Gi Byun^1*^

^1^Division of Electronics, Information and Communication Engineering, Kangwon National University (Samcheok), Kangwon-do 24341, Republic of Korea

^2^Laboratory of Nanoscale Characterization & Environmental Chemistry, Department of Chemistry, College of Natural Sciences, Hanyang University, Seoul 04763, Republic of Korea

*Correspondence: [byun@kangwon.ac.kr](mailto:byun@kangwon.ac.kr)

Supporting information of this manuscript includes:
- Supplementary Table S1. PChem score criteria.

- Supplementary Table S2. The categorical values of nominal attributes.

Supplementary Table S1. PChem score criteria.

| **Attribute** | **Criteria** | | **Score** |
| --- | --- | --- | --- |
| Core size | Data source | - Experimentally measured by the authors | 3 |
|  |  | - Adapted from manufacturers’ specifications | 2 |
|  |  | - Adapted from other references using the same nanomaterials and experimental conditions | 1 |
|  |  | - No data | 0 |
|  | Data method | - TEM | 2 |
|  |  | - Estimated from specific surface area  - Other methods (e.g., SEM/AFM) | 1 |
|  |  | - No information | 0 |
| Hydrodynamic size | Data source | - Experimentally measured by the authors | 3 |
|  |  | - Adapted from manufacturers’ specifications | 2 |
|  |  | - Adapted from other references using the same nanomaterials and experimental conditions | 1 |
|  |  | - No data | 0 |
|  | Data method | - DLS/NTA | 2 |
|  |  | - Other methods | 1 |
|  |  | - No information | 0 |
| Surface charge | Data source | - Experimentally measured by the authors | 3 |
|  |  | - Adapted from manufacturers’ specifications | 2 |
|  |  | - Adapted from other references using the same nanomaterials and experimental conditions | 1 |
|  |  | - No data | 0 |
|  | Data method | - Zeta potential | 2 |
|  |  | - Other methods | 1 |
|  |  | - No information | 0 |
| Specific surface area | Data source | - Experimentally measured by the authors | 3 |
|  |  | - Adapted from manufacturers’ specifications | 2 |
|  |  | - Adapted from other references using the same nanomaterials and experimental conditions | 1 |
|  |  | - No data | 0 |
|  | Data method | - BET | 2 |
|  |  | - Estimated from core size  - Other methods | 1 |
|  |  | - No information | 0 |

TEM: Transmission Electron Microscopy; SEM: Scanning Electron Microscopy; AFM: Atomic Force Microscopy; XRD: X-Ray Diffraction; DLS: Dynamic Light Scattering; NTA: Nanoparticle Tracking Analysis; BET: Brunauer-Emmett-Teller method.

Supplementary Table S2. The categorical values of nominal attributes.

| **No.** | **Assay method** | **Cell name** | **Cell species** | **Cell origin** | **cell type** |
| --- | --- | --- | --- | --- | --- |
| 1 | MTT | HCMEC | Human | Lung | Cancer |
| 2 | Alamar blue | A549 | Hamster | Bone | Normal |
| 3 | LDH | MeT-5A | Mouse | Blood |  |
| 4 | NRU | BEAS-2B |  | Colon |  |
| 5 | Annexiv V/PI staining | SH-SY5Y |  | Liver |  |
| 6 | ATP | MCF-10A |  | Nose |  |
| 7 | MTS | WI-38 |  | Mesothelium |  |
| 8 | CyQuant Assay | Chang Liver |  | Breast |  |
| 9 |  | L-02 |  |  |  |
| 10 |  | V79-4 |  |  |  |
| 11 |  | RAW264.7 |  |  |  |
| 12 |  | SW480 |  |  |  |
| 13 |  | Caco-2 |  |  |  |
| 14 |  | Human olfactory neurosphere-derived cells |  |  |  |
